# Renal Leukocyte Chemotactic Factor 2 (ALECT2) Amyloidosis With Concurrent IgA Nephropathy: A Case Report and Literature Review

**DOI:** 10.7759/cureus.83192

**Published:** 2025-04-29

**Authors:** Qian Gong, Lutao Gao, Hongxia Li, Rongzheng Zhong, Yanhong Huo

**Affiliations:** 1 Health Sciences Center, Peking University, Beijing, CHN; 2 Department of Health Economics, Seventh Medical Center of Chinese PLA General Hospital, Beijing, CHN; 3 Department of Pathology, Seventh Medical Center of Chinese PLA General Hospital, Beijing, CHN; 4 Department of Nephrology, Seventh Medical Center of Chinese PLA General Hospital, Beijing, CHN; 5 Department of Nephrology, First Medical Center of Chinese PLA General Hospital, Beijing, CHN

**Keywords:** alect2, amyloidosis, case report, chronic kidney disease, iga nephropathy, leukocyte chemotactic factor 2 amyloidosis

## Abstract

Leukocyte chemotactic factor 2 (ALECT2) amyloidosis is a rare form of renal amyloidosis, typically presenting with chronic kidney disease and variable degrees of proteinuria. Cases of concurrent ALECT2 amyloidosis and IgA nephropathy are exceedingly rare, with limited descriptions of their clinical and pathological features in the literature. Here, we report a 61-year-old Chinese woman who presented with symmetrical lower limb edema and microscopic hematuria, without nephrotic syndrome or significant proteinuria. Renal biopsy revealed diffuse interstitial amyloid deposition and coexisting IgA nephropathy. Laser microdissection combined with mass spectrometry (LMD/MS) confirmed the presence of LECT2 amyloid protein. The patient was diagnosed with ALECT2 amyloidosis with concurrent IgA nephropathy. She was treated with sodium-glucose transport protein 2 (SGLT-2) inhibitors, and her renal function stabilized at a six-month follow-up. Combining immunological techniques and LMD/MS is recommended for the diagnosis of renal ALECT2 amyloidosis.

## Introduction

Leukocyte chemotactic factor 2 (ALECT2) amyloidosis is the third most common form of renal amyloidosis but remains rare overall [1]. Unlike the more common immunoglobulin light chain (AL) amyloidosis and amyloid A (AA) amyloidosis, ALECT2 amyloidosis rarely involves extrarenal organs, nephrotic syndrome is uncommon, and proteinuria tends to be variable [2,3]. It is characterized by diffuse interstitial amyloid deposition [2]. ALECT2 amyloidosis can coexist with other renal diseases, though cases with IgA nephropathy are rare. Congo red staining, immunological techniques, and laser microdissection and mass spectrometry (LMD/MS) are essential for accurate diagnosis, especially in cases with minimal amyloid deposits [4]. Here, we report an unusual case of ALECT2 amyloidosis coexisting with IgA nephropathy, with an indolent onset and mild progression of renal impairment.

## Case presentation

A 61-year-old Chinese woman developed symmetrical pitting edema in both lower limbs in February 2024 following an upper respiratory tract infection. This was accompanied by fever, with a maximum temperature of 38°C, which resolved spontaneously. She did not take antibiotics or nonsteroidal anti-inflammatory drugs (NSAIDs). There was no foamy urine or gross hematuria. In July 2024, over the 10 days before admission, her lower limb edema progressively worsened. On physical examination, bilateral lower limb edema was noted, with no other abnormalities. She did not have a significant past medical history or a family history of hereditary kidney disease.

In February 2024, laboratory testing revealed a blood urea nitrogen (BUN) level of 7.84 mmol/L and serum creatinine of 162 μmol/L. Urinalysis demonstrated protein (+) and blood (+++) (Table 1). In July 2024, at the time of presentation, urinalysis showed no proteinuria but revealed red blood cells (RBCs) in the urinary sediment at 12.7/HPF. Laboratory studies revealed the following results: BUN, 10.5 mmol/L; serum creatinine, 131 μmol/L; serum homocysteine, 28.8 μmol/L; cystatin C, 1.54 mg/L; urine α1-microglobulin, 3.18 mg/L; urine β2-microglobulin, 3.56 mg/L; IgA, 4.77 g/L; and 24-hour urine total protein of 340 mg/day (Table 2). Serum immunofixation electrophoresis detected no monoclonal protein bands. Other serologic tests, including hepatitis B markers, antinuclear antibody (ANA) profile, antineutrophil cytoplasmic antibody (ANCA) panel, thyroid function tests, tumor markers, and complement levels, were all within normal limits.

**Table 1:** Laboratory results in February 2024 *Taken from the urine sample

Test	Result	Normal range
Blood urea nitrogen	7.84	3.1-8.8 mmol/L
Serum creatinine	162	41-81 μmol/L
Protein*	+	-
Blood*	+++	-

**Table 2:** Laboratory results in July 2024 *Taken from the urine sample

Test	Result	Normal range
Blood urea nitrogen	10.5	2.86-8.21 mmol/L
Serum creatinine	131	41-81 μmol/L
Serum homocysteine	28.8	7-14 μmol/L
Cystatin C	1.54	0.55-1.05 mg/L
Urine α1-microglobulin	3.18	0-1.25 mg/dL
Urine β2-microglobulin	3.56	0.1-0.3 mg/L
IgA	4.77	0.4-3.3 g/L
Protein*	-	<0.15 g/L
Red blood cells*	12.7	0-3.0 /HPF
24-hour urine total protein*	340	<120 mg/24h

**Figure 1:**
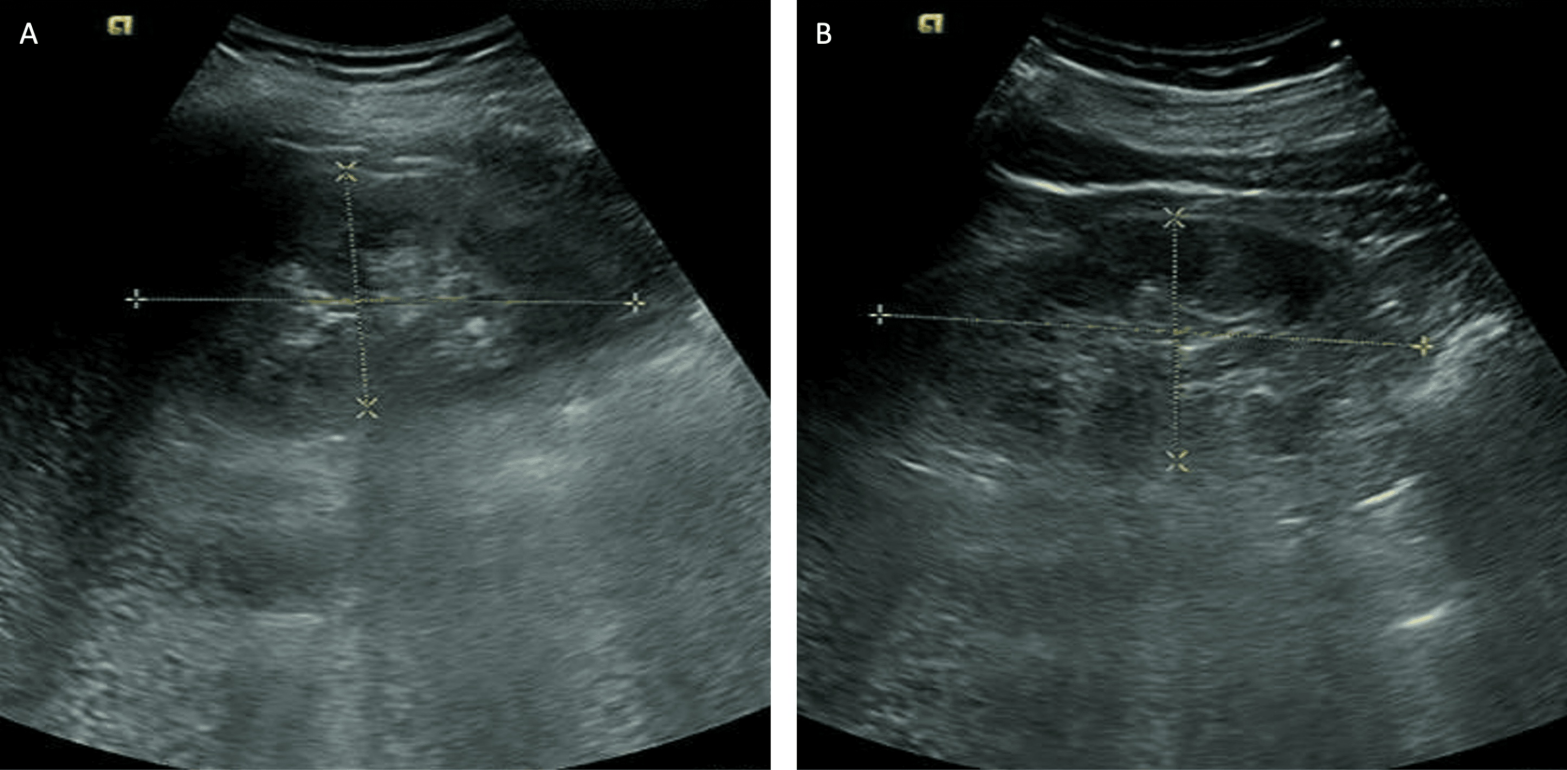
Renal ultrasound revealed diffuse lesions in both kidneys. (A) The right kidney. (B) The left kidney

Renal ultrasound revealed diffuse lesions in both kidneys, with unclear differentiation between the renal cortex and medulla (Figure 1). Cardiac and abdominal ultrasounds showed no abnormalities.

**Figure 2:**
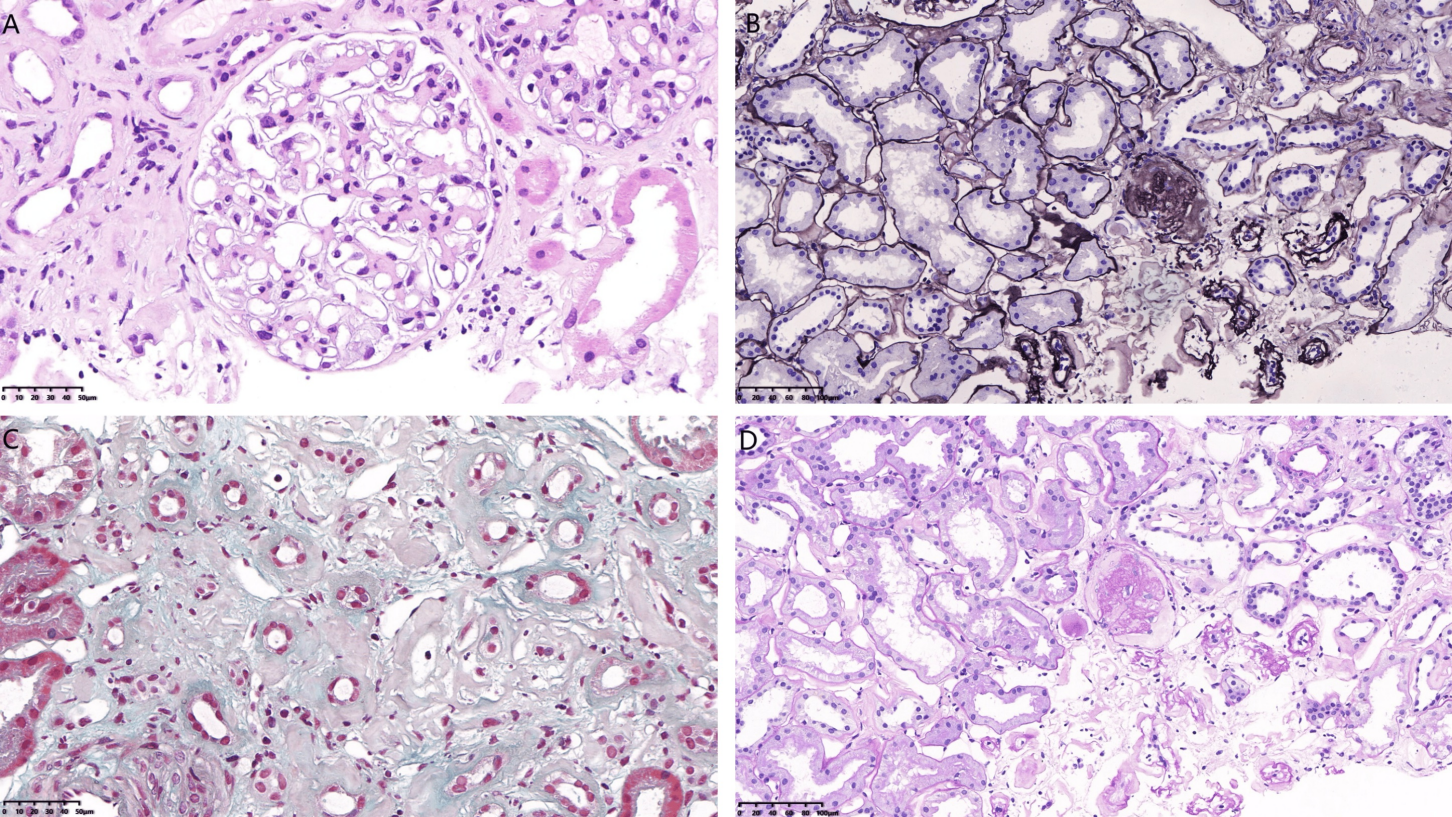
Light microscopy findings. (A) Hematoxylin and eosin staining (X400). (B) PASM staining (X100). (C) Masson’s trichrome staining (X400). (D) PAS staining (X100) PASM: Periodic Schiff-methenamine silver; PAS: periodic acid-Schiff

**Figure 3:**
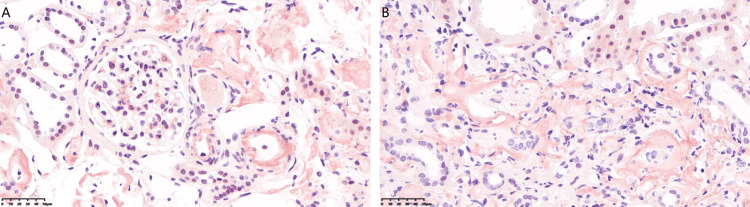
Focal deposition of congophilic amyloid material in glomerular mesangium, renal interstitium, and arterial walls. (A) Glomerulus and arteriole (X400). (B) Tubulointerstitial area (X400)

**Figure 4:**
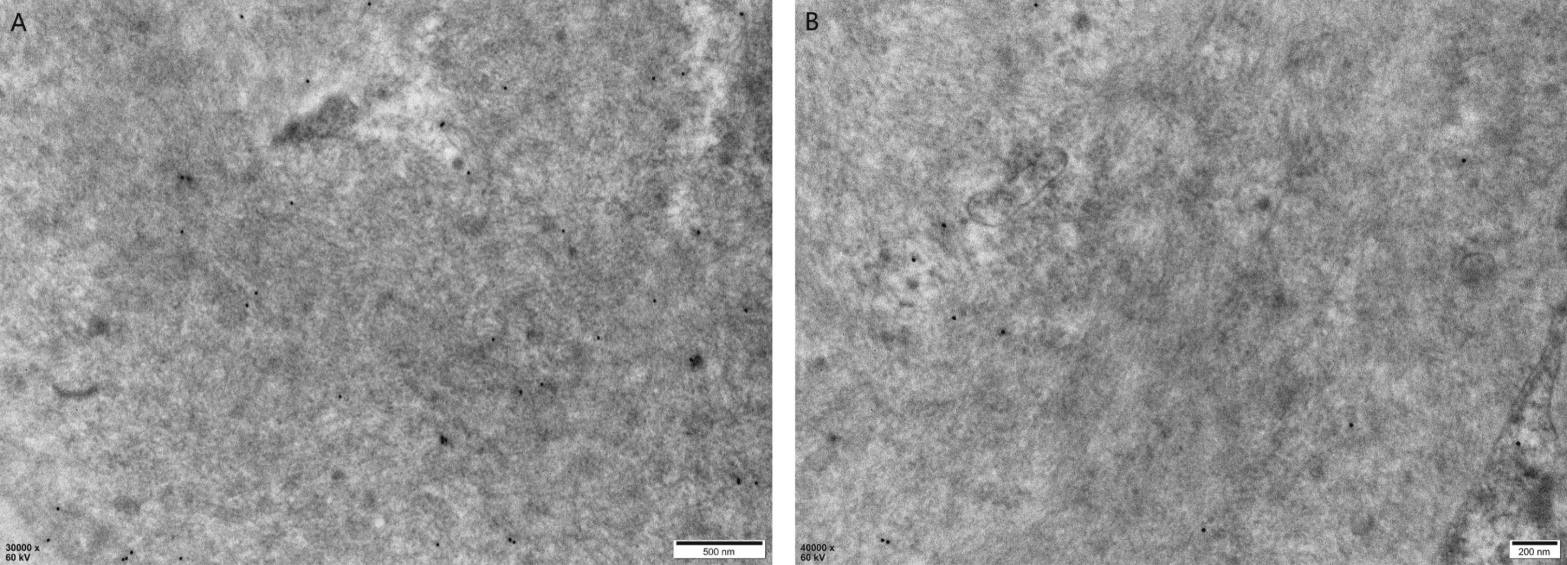
Immunoelectron microscopy: κ (+), λ (±). (A) κ immunoelectron microscopy (X30,000). (B) λ immunoelectron microscopy (X40,000)

Renal biopsy revealed nine glomeruli, one of which showed ischemic sclerosis, while the others exhibited uniform deposition of abnormal proteins without mesangial matrix expansion. Tubulointerstitial damage was prominent, characterized by vacuolar and granular degeneration of tubular epithelial cells, multifocal tubular atrophy, amyloid stain-positive protein casts, interstitial fibrosis, and lymphocyte and monocyte infiltration. Patchy amyloid deposits were also observed in the interstitium. Small arteries showed intimal fibrous hyperplasia, luminal narrowing, and segmental hyalinosis (Figure 2). Congo red staining revealed amyloid deposits in the glomerular mesangium, renal interstitium, and arterial walls (Figure 3). Immunofluorescence showed IgA++ and C3++ deposits in the mesangium, while IgG, IgM, and C1q were negative. Immunoelectron microscopy did not detect leukocyte chemotactic factor 2 (LECT2) protein, and κ(+) and λ(±) light chains were identified, which closely resemble AL amyloidosis (Figure 4).

**Figure 5:**
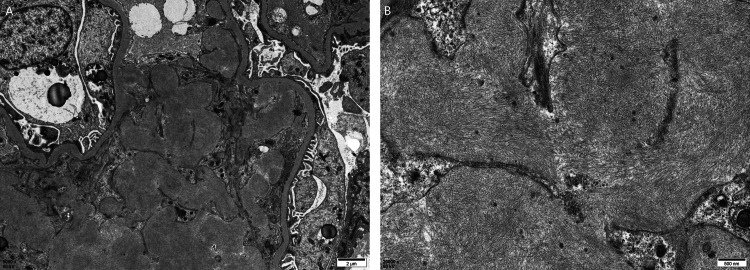
Electron microscopy: (A) (X5,000). (B) (X20,000)

**Figure 6:**
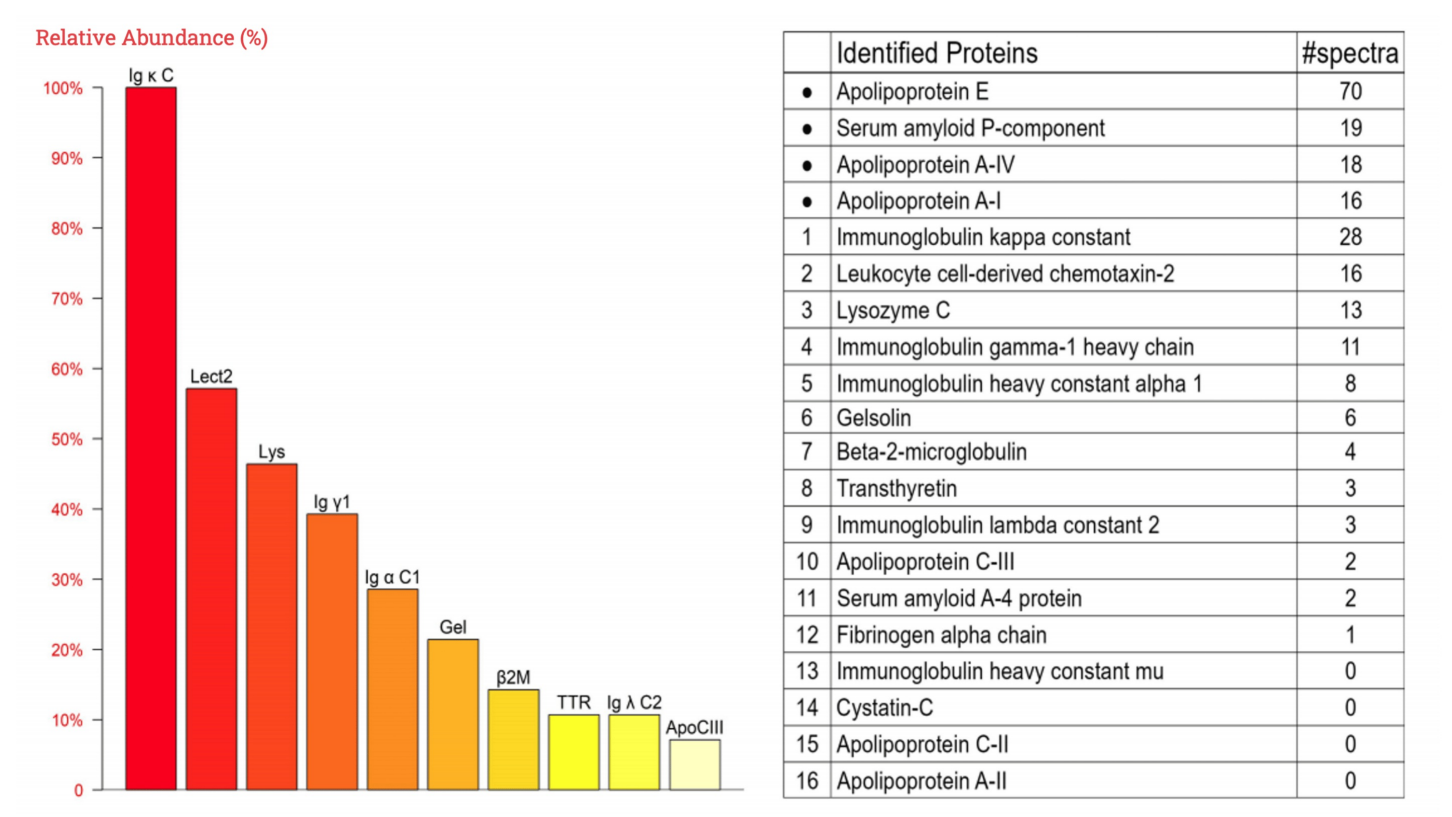
Laser microdissection and mass spectrometry (LMD/MS): high-abundance apolipoproteins (Apo) were detected, confirming the presence of amyloid deposition. LECT2 protein was identified, supporting the diagnosis of the ALECT2 subtype LECT2: leukocyte chemotactic factor 2; ALECT2: leukocyte chemotactic factor 2 amyloidosis

Electron microscopy showed randomly arranged, nonbranching fibrils (~10 nm in diameter) within the glomeruli, along with electron-dense deposits in the mesangial and paramesangial areas, as well as similar fibrillar deposits in the interstitium and small arteries (Figure 5). Laser microdissection of Congo red-positive areas from formalin-fixed paraffin-embedded (FFPE) sections, followed by liquid chromatography-tandem mass spectrometry, confirmed the presence of LECT2 amyloid protein (Figure 6). Therefore, the patient was finally diagnosed with ALECT2 amyloidosis with concurrent IgA nephropathy. Genetic analysis revealed no mutations in the LECT2 gene in this case.

The patient was treated with sodium-glucose cotransporter 2 (SGLT-2) inhibitors due to intolerance to angiotensin receptor blockers (ARBs). At the six-month follow-up, serum creatinine was 117 μmol/L, with a urine microalbumin-to-creatinine ratio of 84.3 mg/g, no proteinuria, and 0-1 red blood cells/HPF.

## Discussion

Amyloidoses are a rare group of diseases caused by the extracellular deposition of misfolded protein fibrils, leading to progressive organ dysfunction [5]. To date, 42 proteins are recognized as amyloidogenic in humans [6]. Among these, AL, AA, and ALECT2 amyloidosis are the three most common types of renal amyloidosis [1].

The etiology of ALECT2 amyloidosis remains unclear and may be associated with LECT2 protein deposition in localized tissues. LECT2 is a multifunctional protein involved in neutrophil chemotactic activity, cell growth, and tissue repair following injury [7]. Although synthesized predominantly in the liver, LECT2 is also expressed in various other cell types across multiple organs, including vascular endothelial cells, smooth muscle cells, and epithelial cells such as renal tubular epithelial cells [8]. No mutations in the LECT2 gene have been identified in patients with ALECT2 amyloidosis, but most of these patients are homozygous for the G allele encoding valine at position 40 in the mature protein [1,7]. ALECT2 amyloidosis demonstrates an ethnic or geographic bias, with a particularly high prevalence observed in Hispanic patients in the southwestern United States [9]. In China, single-center data from Shanxi Province revealed that ALECT2 amyloidosis accounted for as much as 12.4% of cases, significantly higher than other reports from other regions in the country [10].

Unlike AL and AA amyloidosis, ALECT2 rarely involves extrarenal organs and is generally associated with superior patient survival. Nephrotic syndrome is uncommon, and proteinuria tends to be variable, as patients may present with gradually worsening renal function without significant proteinuria [2,3]. ALECT2 amyloidosis is characterized by diffuse interstitial deposition with or without mesangial and vascular involvement [1,11]. In contrast, AL and AA amyloidosis primarily involve the glomeruli and small arterial walls.

ALECT2 amyloidosis can coexist with other renal diseases; however, cases coexisting with IgA nephropathy are relatively rare. In a multicenter study involving 72 patients diagnosed with renal ALECT2 amyloidosis, 19 patients (26.4%) were found to have additional kidney diseases on biopsy. The most common coexisting condition was diabetic glomerulosclerosis (10 cases, 13.9%), followed by IgA nephropathy (five cases, 6.9%) [8]. Another study reported 40 ALECT2 amyloidosis cases, 10 of which had additional histological diagnoses. The most frequent was arterionephrosclerosis (five cases, 14%), followed by diabetic glomerulosclerosis (two cases, 6%) [12].

Studies from China have shown that membranous nephropathy (MN) is the most common renal disease coexisting with ALECT2 amyloidosis. In a single-center study [13], among seven patients diagnosed with ALECT2 amyloidosis, four were also found to have MN. Another single-center study reported 15 cases of ALECT2 amyloidosis, of which four coexisted with MN and two with IgA nephropathy [10].

A previously reported case of renal ALECT2 amyloidosis combined with IgA nephropathy presented with nephrotic syndrome, characterized by more prominent glomerular lesions and relatively mild interstitial involvement [14]. In contrast, our case had an extremely insidious onset, presenting only with edema and microscopic hematuria, without clinical features of nephrotic syndrome. Urine immunoelectrophoresis and clinical findings suggested tubular injury, which was further confirmed by renal biopsy showing predominantly tubular damage. Compared to cases of isolated ALECT2 amyloidosis, the overall deposition of amyloid in this case was significantly lower, and Congo red staining did not show a strongly positive result, potentially indicating that the renal biopsy was performed at an early stage of amyloid deposition.

The coexistence of ALECT2 amyloidosis with other glomerular diseases remains poorly understood, as it is unclear whether this represents a pathological association or a coincidence. In our case of ALECT2 amyloidosis coexisting with IgA nephropathy, mesangial proliferation caused by the glomerular disease, along with tubular atrophy and interstitial fibrosis, to some extent, increases the difficulty of identifying amyloid deposits. Failure to carefully examine light microscopy findings often leads to missed diagnoses of amyloidosis, which underscores the importance of performing routine Congo red staining.

Similar to IgA nephropathy, there are currently no laboratory tests available to diagnose ALECT2 renal amyloidosis. Renal biopsy remains essential for diagnosis, particularly in cases of unexplained progressive renal function decline without hematuria or proteinuria. Notably, LMD/MS is critical for the diagnosis of ALECT2 amyloidosis. Previous studies have emphasized that immunohistochemistry alone may be insufficient for diagnosing ALECT2 amyloidosis in cases with weakly positive staining. Combining immunohistochemistry with LMD/MS can help ensure accurate diagnosis and minimize potential errors [3]. Another study suggests that LMD/MS alone may be insufficient for diagnosing ALECT2 amyloidosis in cases with minimal amyloid deposits, whereas immunoelectron microscopy (IEM) proves to be a more sensitive method for early diagnosis and amyloid typing, including ALECT2 amyloidosis [13]. In our case, immunoelectron microscopy revealed κ (+) and λ (±) staining without the detection of LECT2 protein, closely resembling AL amyloidosis. However, the clinical presentation differed significantly from AL amyloidosis, and the diagnosis of ALECT2 was ultimately confirmed through LMD/MS. Therefore, we recommend combining immunological techniques with LMD/MS as essential tools for the accurate diagnosis of ALECT2 amyloidosis, especially in cases initially diagnosed as AL amyloidosis, where chemotherapy is being considered.

Currently, there is no specific treatment for ALECT2 renal amyloidosis. Its natural progression is relatively slow, with an average glomerular filtration rate (GFR) decline of 4.2 mL/min/year [15]. Management is primarily supportive, and kidney transplantation may be considered in end-stage cases [3].

## Conclusions

Renal ALECT2 amyloidosis is a rare condition often prone to being missed or misdiagnosed, and its coexistence with IgA nephropathy is even rarer. This case highlights the need for comprehensive pathological evaluation, combining immunological techniques with LMD/MS, to ensure accurate diagnosis and avoid inappropriate treatments. Further studies are needed to improve our understanding of the pathological interplay between ALECT2 amyloidosis and coexisting renal diseases, as well as to explore potential therapeutic strategies tailored to these conditions.

## Appendices

Informed consent was obtained in both written and verbal formats from the patient to publish this case report and any accompanying images.
